# The Non-phosphorylating Glyceraldehyde-3-Phosphate Dehydrogenase GapN Is a Potential New Drug Target in *Streptococcus pyogenes*

**DOI:** 10.3389/fmicb.2022.802427

**Published:** 2022-02-15

**Authors:** Philip Eisenberg, Leon Albert, Jonathan Teuffel, Eric Zitzow, Claudia Michaelis, Jane Jarick, Clemens Sehlke, Lisa Große, Nicole Bader, Ariane Nunes-Alves, Bernd Kreikemeyer, Hermann Schindelin, Rebecca C. Wade, Tomas Fiedler

**Affiliations:** ^1^Institute of Medical Microbiology, Virology, and Hygiene, Rostock University Medical Centre, Rostock, Germany; ^2^Rudolf Virchow Center for Integrative and Translational Bioimaging, University of Würzburg, Würzburg, Germany; ^3^Molecular and Cellular Modeling Group, Heidelberg Institute for Theoretical Studies, Heidelberg, Germany; ^4^Center for Molecular Biology (ZMBH), DKFZ-ZMBH Alliance, Heidelberg University, Heidelberg, Germany; ^5^Interdisciplinary Center for Scientific Computing (IWR), Heidelberg University, Heidelberg, Germany

**Keywords:** X-ray crystallography, homology modeling, computational docking, PNA (peptide nucleic acid), NADPH, *Streptococcus pyogenes*, drug target, GapN

## Abstract

The strict human pathogen *Streptococcus pyogenes* causes infections of varying severity, ranging from self-limiting suppurative infections to life-threatening diseases like necrotizing fasciitis or streptococcal toxic shock syndrome. Here, we show that the non-phosphorylating glyceraldehyde-3-phosphate dehydrogenase GapN is an essential enzyme for *S. pyogenes*. GapN converts glyceraldehyde 3-phosphate into 3-phosphoglycerate coupled to the reduction of NADP to NADPH. The knock-down of *gapN* by antisense peptide nucleic acids (asPNA) significantly reduces viable bacterial counts of *S. pyogenes* laboratory and macrolide-resistant clinical strains *in vitro*. As *S. pyogenes* lacks the oxidative part of the pentose phosphate pathway, GapN appears to be the major NADPH source for the bacterium. Accordingly, other streptococci that carry a complete pentose phosphate pathway are not prone to asPNA-based *gapN* knock-down. Determination of the crystal structure of the *S. pyogenes* GapN apo-enzyme revealed an unusual cis-peptide in proximity to the catalytic binding site. Furthermore, using a structural modeling approach, we correctly predicted competitive inhibition of *S. pyogenes* GapN by erythrose 4-phosphate, indicating that our structural model can be used for *in silico* screening of specific GapN inhibitors. In conclusion, the data provided here reveal that GapN is a potential target for antimicrobial substances that selectively kill *S. pyogenes* and other streptococci that lack the oxidative part of the pentose phosphate pathway.

## Introduction

In the last decades, a rapid increase in antibiotic resistances of pathogenic bacteria has been observed (World Health Organization [WHO], 2014). This alarming development is exacerbated by the so-called *discovery void* of new antimicrobial substances during the last 30 years (Silver, 2011). Infections with antibiotic-resistant bacteria represent a severe problem in different ways. First, the prognosis for patients infected with multiresistant bacteria is unfavorable. Second, infections with multiresistant bacteria represent a high financial burden for the public healthcare systems due to long hospitalizations of patients requiring complex therapeutic measures (Resch et al., 2009; Roca et al., 2015; Thabit et al., 2015).

Usually, antibiotics are not specific for a particular bacterial species or even a genus. They normally not only kill the targeted pathogenic bacteria, but also severely interfere with (part of) the commensal flora (Willing et al., 2011). Hence, there is an urgent need for the development of new and efficient antibiotics targeting specifically those bacteria that need to be eradicated.

*S. pyogenes* (group A streptococcus, GAS) is one of the most important gram-positive pathogens. It usually first colonizes the skin or mucosal membranes of the upper respiratory tract and induces local purulent infections (e.g., tonsillitis, pharyngitis, impetigo), however, toxin-mediated, invasive or systemic diseases (e.g., scarlet fever, toxic-shock-like syndrome, necrotizing fasciitis) as well as autoimmune-sequelae (rheumatic fever, rheumatic heart disease) can occur (Walker et al., 2014). The estimated global annual burden of *S. pyogenes* diseases comprises about 616 million cases of tonsillitis/pharyngitis, 111 million cases of skin infections and 0.5 million deaths due to severe infections (Carapetis et al., 2005).

Tonsillitis caused by *S. pyogenes* (strep throat) is a frequent reason for the prescription of antibiotics outside hospitals. These infections are usually treated with penicillin for 7–10 days or with macrolides for 3 days (Bisno et al., 2002; Shulman et al., 2012). According to recent studies, basic penicillins and macrolides are two of the most frequently prescribed antibiotics in Germany (20 and 15% of patients given antibiotic treatment, respectively; Centers for Disease Control and Prevention [CDC], 2019b) (Holstiege et al., 2020) and in the United States (23.4 and 15.9% of outpatients given antibiotic treatment, respectively). While penicillin resistance has not yet been observed in *S. pyogenes*, macrolide resistant strains are frequently isolated from patients. In the respective studies, up to 38% of the clinical isolates were erythromycin-resistant (Rubio-Lopez et al., 2012). Furthermore, penicillin treatment frequently fails, which might be associated with either intracellular persistence of the bacteria or the formation of impenetrable biofilms (Facinelli et al., 2001; Baldassarri et al., 2006).

In the “2019 Antibiotics Resistance Threat Report” of the Centers for Disease Control and Prevention of the United States the erythromycin-resistant *S. pyogenes* is classified as a concerning threat that requires careful monitoring and preventative action as a result of the rising number of resistant strains isolated (Centers for Disease Control and Prevention [CDC], 2019a). Erythromycin resistance in *S. pyogenes* is associated with the ability of the bacteria to enter human respiratory cells, thereby escaping extracellular antibiotics such as penicillin (Facinelli et al., 2001).

Another unfavorable observation is the occurrence of *S. pyogenes* isolates carrying a specific mutation in the penicillin-binding protein PBP2x, leading to a reduced β-lactam antibiotic sensitivity in the last years (Musser et al., 2020; Southon et al., 2020; Vannice et al., 2020). Such mutations might be the starting point for resistance development (Southon et al., 2020).

Considering all of the above, it is of paramount importance to search for new treatment strategies against *S. pyogenes* infections.

The central metabolism of bacteria is a potential target for the development of new antimicrobial substances. New targets for antimicrobial substances should be metabolic processes that (i) are not present or not essential in human cells, and (ii) are not present or not essential in the majority of the species of the physiological flora.

The non-phosphorylating glyceraldehyde-3-phosphate dehydrogenase GapN has been predicted to be an essential enzyme in *S. pyogenes* M49 based on gene essentiality analyses via transposon mutant libraries (Le Breton et al., 2015). GapN catalyzes the irreversible oxidation of glyceraldehyde 3-phosphate (G3P) to 3-phosphoglycerate (3-PG) while reducing NADP^+^ to NADPH (Habenicht, 1997). Hence, it provides NADPH that is usually generated in the oxPPP, which is missing in *S. pyogenes* and other streptococci (Crow and Wittenberger, 1979). Furthermore, since this reaction does not consume inorganic phosphate, it is hypothesized that GapN maintains glycolysis under low-phosphate conditions (Levering et al., 2012). Therefore, GapN is a promising target for specific inhibition of growth of *S. pyogenes* and other streptococci, since GapN-like enzymes are most likely not essential for bacteria possessing a complete pentose phosphate pathway. Iddar et al. (2003) previously investigated physicochemical and catalytic properties of the *S. pyogenes* GapN and showed that (a) the enzyme is active in a pH range between 5 and 13 with its pH optimum at 8.5, (b) the enzyme is stable at temperatures between 15 and 40°C but loses activity at temperatures above 45°C, and (c) that the enzyme oxidizes D- and DL-G3P using NADP as a cofactor with k_*m*_ values of 0.666 mM for G3P and 0.385 mM for NADP (Iddar et al., 2003).

Antisense peptide nucleic acids (asPNAs) are useful tools for interfering with specific target molecules. PNAs have peptide backbones decorated with nucleobases capable of base pairing. Because of their chemical composition, PNAs are inaccessible to nucleases and proteases. However, PNAs do not readily enter the target cell. This problem can be overcome by cell penetrating peptides (CPPs) fused to the PNA. For *S. pyogenes*, the (RXR)_4_XB peptide has been shown to allow sufficient entry of PNAs (directed against the gyrase subunit A gene *gyrA*) into the bacteria to accomplish growth inhibition (Herce et al., 2009; Patenge et al., 2013; Barkowsky et al., 2019).

Here, we describe the application of (RXR)_4_XB-fused *gapN*-asPNAs for growth inhibition of *S. pyogenes* laboratory strains and macrolide-resistant clinical isolates. Our data support the hypothesis that the essentiality of GapN is due to the lack of NADPH in the absence of this enzyme in streptococci lacking the oxPPP. Accordingly, GapN represents a promising target for new antibiotic strategies against *S. pyogenes* by cutting off the NADPH supply of the bacteria.

## Materials and Methods

### Bacterial Strains and Culture Conditions

*S. pyogenes* strains and their characteristics are listed in Table 1. Furthermore, *S. cristatus* DSM8249 and *S. dysgalactiae* ssp. *equisimilis* ATCC 12394 were used in this study. Generally, streptococci were grown in Todd-Hewitt medium supplemented with 0.5% (w/v) yeast extract (THY medium) at 37°C under a 5% CO_2_ enriched atmosphere, except for *S. cristatus*, which was grown in Brain Heart Infusion (BHI) medium. For growth- and phosphate-dependent GapN activity analyses, *S. pyogenes* and *S. cristatus* were grown in chemically defined medium for lactic acid bacteria (CDM-LAB) as previously described (Fiedler et al., 2011) but with 0.5 g/l arginine instead of 0.125 g/l. While CDM-LAB contains 42 mM phosphate, in the CDM-LAB low phosphate variant, the phosphate concentration was reduced to 1 mM.

**TABLE 1:** Clinical *S. pyogenes* isolates and their characteristics (Koller et al., 2010).

Strain	Class of infection	Antibiotic resistance	*emm* type	Specific inhibition by gapN asPNA (μM)^a^
591	Skin	–	49	≥ 1
HRO-K-021	Throat	E, L	12	≥ 3
HRO-K-033	Throat	E	4	≥ 3
HRO-K-071	Throat	E, T	77	≥ 3
HRO-K-075	Throat	E	81	≥ 4
HRO-K-094	Throat	E	1	≥ 2
HRO-K-100	Throat	E, T	58	≥ 2
HRO-K-177	Invasive	E, L	28	≥ 3

*E, erythromycin, L, levofloxacin, T, tetracycline.*

*^a^Minimum concentration with a significant reduction in colony-forming units compared to scrPNA-treated controls in the kill assay (p < 0.05, n = 4, Mann-Whitney U-test).*

### Peptide Nucleic Acids

Each PNA was fused to the cell penetrating peptide (RXR)_4_XB (R—arginine, X—6-aminohexanoic acid, B—beta alanine), which has previously been shown to work efficiently in *S. pyogenes* M49 (Barkowsky et al., 2019). High-performance liquid chromatography purified and matrix-assisted laser desorption/ionization mass spectrometry analyzed CPP-fused PNAs were obtained from Peps4LS GmbH (Heidelberg, Germany). Results of the quality controls performed by Peps4LS GmbH for each PNA used in this study are shown as Supplementary Table 1. Sequences and characteristics of the PNAs used in this study are listed in Table 2. Antisense-PNA were designed to bind to the start codon region of their target gene. All scrambled controls were designed by shuffling the bases of the specific PNA in random order. Each scrambled PNA was blasted (short sequence algorithm) with the respective genome to make sure that the scrambled PNA are not complementary to the start codon region of any gene in the respective genome.

**TABLE 2:** PNA sequences.

Species	Target	CPP	PNA sequence
*S. dysgalactiae* ssp. *equisimilis*	*gapN*	(RXR)_4_XB	TTGTCAACGT
	*gyrA*	(RXR)_4_XB	TGCATTTAAG
	- (scr_*gapN*) - (scr_*gyrA*)	(RXR)_4_XB (RXR)_4_XB	TCAGTCAGTT ATTAGACTGT
*S. pyogenes*	*gapN*	(RXR)_4_XB	TTGCCAACGT
	*gyrA*	(RXR)_4_XB	TGCATTTAAG
	- (scr_*gapN*)	(RXR)_4_XB	CATGTGCTAC
*S. cristatus*	*gapN*	(RXR)_4_XB	CACGTGACAC
	*gyrA*	(RXR)_4_XB	CTTGCATTAA
	- (scr_*gapN*) - (scr_*gyrA*)	(RXR)_4_XB (RXR)_4_XB	CCCCGGAAAT TAGTACTACT

### Kill Assays

Bacterial susceptibility to PNA was tested by using the method previously published by Barkowsky et al. (2019). The protocol was adopted for *S. cristatus* and *S. dysgalactiae* ssp. *equisimilis*. THY or BHI overnight cultures of the respective strains were dissolved in PBS containing either 20% (v/v) BHI (*S. pyogenes*), 15% BHI (*S. cristatus*), 10% BHI (*S. dysgalactiae* ssp. *equisimilis*) to 10^5^ CFU/ml. Antisense PNA or the respective scrambled control PNA were mixed 1:9 with the bacterial suspension and incubated for 6 h at 37°C in a rotator. As a control, bacteria were incubated under the same conditions in the absence of PNA. Subsequently, viable counts were determined by plating serial dilutions on THY or BHI agar plates.

### Growth Experiments

To analyze the transcription and enzyme activity at different growth phases, CDM-LAB or CDM-LAB low phosphate was inoculated 1:20 with bacteria of THY or BHI overnight cultures suspended in 0.9% (w/v) NaCl and incubated at 37°C, 5% CO_2_. The bacterial growth was controlled by measuring the optical density at 600 nm. *S. pyogenes* samples were collected in the exponential (OD_600_ _nm_ = 0.35–0.45, 2.5 h after inoculation), transition (OD_600_ _nm_ = 0.9–1.1, 4 h after inoculation) and stationary phase (OD_600_ _nm_ = 1.55–1.65, 7 h after inoculation), whereas *S. cristatus* samples were collected in the exponential (OD_600_ _nm_ = 0.35–0.5, 2.5 h after inoculation) and stationary phase (OD_600_ _nm_ = 2.55–2.65 (42 mM phosphate), OD_600_ _nm_ = 1.65–1.9 (1 mM phosphate), 6.5 h after inoculation). Subsequently, the samples were spun down at 4,000 × *g*, 4°C for 10 min. The pellets were flash frozen in liquid nitrogen and stored at –20°C.

### Quantitative Real-Time PCR

For qPCR, RNA was isolated from respective samples via phenol-chloroform extraction. For that purpose, bacteria were suspended in 1 ml TRIZOL^®^ reagent and treated in a Precellys 24 homogenizer (Bertin Technologies SAS, Montigny-le-Bretonneux, France) with two cycles of 30 s each at a speed of 6,500 rpm and cooling in between for 2 min on ice. After centrifugation at 16,000 × *g* and 4°C for 5 min the supernatant was mixed with 600 μl acid phenol-chloroform-isoamylalcohol (25:24:1) (APCI) and 20 μl 1 M sodium acetate in a Precellys 24 homogenizer with two cycles of 10 s at a speed of 4,000 rpm, incubated for 5 min at room temperature and centrifuged for 5 min at 5,300 × *g* and 4°C. With the upper, aqueous phase this procedure was repeated three times. Finally, the aqueous phase was mixed with 1 ml pure ethanol at –20°C and incubated for 16-18 h at –20°C and then centrifuged for 60 min at 18,500 × *g* and 4°C. The nucleic acid pellet was washed with 1 ml 75% (v/v) ethanol in DEPC-treated water at –20°C, dried and suspended in 50 μl DEPC-treated water. The remaining DNA in the samples was removed using the TURBO DNA-free™ Kit (Invitrogen, Waltham, MA, United States) and the RNA concentration was determined using a PicoDrop. Fifty nanogram of total RNA were transcribed into cDNA using the First Strand cDNA synthesis kit (Invitrogen, Waltham, MA, United States) following the manufacturer’s instructions. The real-time PCR was carried out with Maxima SYBR Green/ROX qPCR Master Mix (Thermo Scientific, Waltham, MA, United States) on a LightCycler 480 II (Roche, Basel, Switzerland). The 5S rRNA gene transcript was used for normalization. For that purpose the primer pair 5′-TGAGTGTCATTGTGGCAAGAGC-3′/5′-AGAGAATACGACGATGCACAGG-3′ was used. The *gapN* transcript was detected using the primers 5′-GAAGAAGGGCT TCGTATGG-3′ and 5′-AGAACCTGCCAAGTTAACGG-3′. Primer efficiency was tested on genomic *S. pyogenes* M49 DNA before use in reverse transcription reactions. The relative gene expression was determined by the ΔΔCT method.

### Heterologous Expression and Purification of GapN and GapDH

The chromosomal DNA of *S. pyogenes* M49 strain 591 served as the template for PCR amplification of the *gapN* gene (primers 5′-GAGATGAATTCTTGGCAAAACAATATAAAA-3′ and 5′-CAATA-GTTGGATCCATCTGTATAGACTA-3′) and the *gapDH* gene (primers 5′-AGGAAATCAGGATC-CGTAGT TAAAGTTGGT and 5′- CGTTATAACGTCGACTTTAGCAAT TTTTGC-3′) with the Phusion™ High Fidelity PCR Kit (Thermo Scientic, Waltham, MA, United States). The resulting *gapDH* PCR fragment was ligated into the pASK-IBA2 vector (IBA GmbH, Göttingen, Germany) system via the *Bam*HI and *Sal*I restriction sites. The *gapN* fragment was ligated into the pASK-IBA6 vector via the *Eco*RI and *Bam*HI restriction sites. *E. coli* DH5α cells were transformed with recombinant vectors. The correct insertion of the PCR product was confirmed by sequencing. For heterologous expression, the recombinant *E. coli* strain was grown in 500 ml LB medium at 37°C under vigorous shaking. At an optical density (600 nm) of about 0.4, expression was induced by addition of anhydrotetracycline (AHT, 0.2 μg/ml). Cells were harvested after overnight shaking at 22°C and pellets were stored at –20°C. For purification of the Strep-tagged proteins, cell pellets were thawed, suspended in 5 ml buffer W (100 mM Tris-HCl pH 8.0, 1 mM EDTA, 150 mM NaCl) and cell disruption was achieved by the FastPrep method with acid-washed glass beads. Two cycles of 30 s each at a speed of 6,500 rpm were applied. In between, samples were cooled on ice. Cell debris was removed by centrifugation at 16,000 × *g* and 4°C for 10 min. Clear supernatants were loaded on StrepTactin sepharose columns (2 ml volume) prewashed with buffer W (2.5 column volumes). Unbound proteins were washed from the column with buffer W (15 column volumes). The protein carrying a StrepTag was eluted from the column in fractions of 1 ml each in buffer E. Buffer E is buffer W supplemented with 10 mM desthiobiotin. The elution fractions were checked for protein purity by SDS-PAGE. Fractions containing pure protein were pooled and dialyzed overnight in PBS or 0.1 M citric acid (pH 6.0) to prepare them for enzymatic assays.

### Measurement of GapN Activity

For the measurement of GapN activity in *S. pyogenes* or *S. cristatus* protein crude extracts, bacteria were subjected to kill assays or growth experiments as described. After 6 h of incubation in the presence of 2 μM *gapN* asPNA, 2 μM scrambled control PNA or in the absence of PNA bacteria were harvested by centrifugation at 16,000 × *g* and 4°C for 10 min. The bacterial pellets were suspended in 500 μl PBS and total protein extracts were prepared in a Precellys 24 homogenizer with two cycles of 30 s each at a speed of 6,500 rpm. Between the cycles the samples were cooled on ice for 2 min. Cell debris was removed by centrifugation at 16,000 × *g* and 4°C for 10 min. Protein concentration in the supernatant was measured using a Qubit protein assay kit following the manufacturer’s instructions. For the measurement of activity of purified GapN, the protein concentration was adjusted to 70 μg/ml. The specific GapN activity was measured as described in Iddar et al. (2003) with modification of the protocol. The assay mixture contained 50 mM Tricine buffer (pH 8.5), 3 mM 2-mercaptoethanol, 10 mM NADP^+^, and 2 mM DL-G3P and 20 μl protein sample in a total volume of 200 μl. The reaction was started by adding DL-G3P. The reduction of NADP^+^ to NADPH was detected by measuring the absorption at 340 nm in a spectrophotometer for up to 60 min. As controls, the assay was carried out without addition of protein sample and without addition of G3P, respectively. GapN activity was calculated with the Lambert-Beer equation and expressed in units per milligram of total protein (U/mg), where one unit is defined as the formation of 1 μmol NADPH per minute. For determination of cofactor specificity, 10 mM NAD^+^ was added to the assay mixture instead of NADP^+^. Potential activating or inhibiting effects on GapN activity were tested by adding 25 mM NaCl, 25 mM KCl, 25 mM NH_4_Cl, 10 mM D-glucose, 10 mM D-fructose, 10 mM sodium pyruvate, 10 mM ATP, 2 mM erythrose 4-phosphate (E4P), 2 mM sedoheptulose 7-phosphate (S7P), 0.4 mM NADH or 0.4 mM NADPH to the assay mixture.

### Measurement of GapDH Activity

The specific GapDH activity was measured as described in Iddar et al. (2003) with modification of the protocol. Purified GapDH was adjusted to a protein concentration of 100 μg/ml. The assay mixture contained 50 mM Tricine buffer (pH 8.5), 3 mM 2-mercaptoethanol, 10 mM NAD^+^, 10 mM PO_4_^3–^, 1 mM DL-G3P and 5 μl protein sample in a total volume of 200 μl. The reaction was started by adding DL-G3P. The reduction of NAD^+^ to NADH was detected by measuring the absorption at 340 nm in a spectrophotometer for up to 120 min. As controls, the assay was carried out without addition of either protein sample or without addition of G3P. Activity was calculated with the Lambert-Beer equation and expressed in units per milligram of total protein (U/mg), where one unit is defined as the formation of 1 μmol NADH per minute. For determination of cofactor specificity, 10 mM NADP^+^ was added to the assay mixture instead of NAD^+^.

### Multi-Angle Light Scattering Analysis of GapN

Size-exclusion chromatography coupled to multi-angle light scattering (SEC-MALS) was used to determine the oligomeric state of GapN in solution. The purified protein was separated at room temperature on a HiLoad 16/600 Superdex 200 pg column (Cytiva, Marlborough, MA, United States) in 1 × PBS (pH 7.4) buffer at a flow rate of 0.5 ml/min. The molecular weight of the eluting proteins was determined using a Dawn Heleos 8 light scattering detector and an Optilab T-rEX refractive index detector (Wyatt Technologies, Santa Barbara, CA, United States). Data were analyzed using the ASTRA software (version 6.1.5.22, Wyatt Technologies, Santa Barbara, CA, United States).

### Homology Modeling of *Streptococcus pyogenes* GapN

Template-based homology modeling of the tetrameric holo-enzyme was conducted using the SWISS-MODEL webserver (Waterhouse et al., 2018) and the sequence of *S. pyogenes* GapN obtained from resequencing (see “Results” section). A holo-structure of GapN from *S. mutans* (PDB 1QI1) (Cobessi et al., 2000) was chosen as the most promising template due to its high sequence-identity of 86%. The molecular structures of the substrate, G3P, and the cofactor, NADP, were inserted into the modeled protein structure by aligning it to the template and subsequently transferring the ligands using PyMol (DeLano, 2002).

### Molecular Docking

Docking of the native substrate (G3P) and two known inhibitors of the template protein from *S. mutans*, E4P and S7P, was conducted using the GLIDE (Friesner et al., 2004) and Autodock4.2 (Morris et al., 2008) molecular docking programs. The GLIDE docking calculations were carried out within the Maestro (Schrödinger, LLC, New York, NY, United States, 2020) environment for molecular modeling (Sastry et al., 2013). After import into the Maestro environment, the protein structure was prepared with the “PrepWizard” utility to generate a protonation state at pH 7.0 ± 3.0, to assign bond orders and to perform a restrained energy minimization with the OPLS2005 force field. Similarly, all ligand structures were prepared using the “LigPrep” program and protonated at a pH of 7.2 ± 0.4. The three-dimensional structures of E4P and S7P were generated with the “2D-sketcher” utility of Maestro (2020). The “standard-precision” mode of GLIDE was used with 1 × van der Waals scaling on the receptor grid and 0.8 × scaling on the ligands. The docking was conducted using default parameters and no additional restraints on the docking optimization. 5 docking poses per ligand were generated and they were assessed by the number of (un-) favorable protein-ligand interactions, the docking scores, and by visual inspection. Docking with Autodock4.2 was conducted using the Autodock Tools GUI (Morris et al., 2008). A Lamarckian Genetic Algorithm was applied with the number of evaluations set to “long.” All other parameters were set to their default values. The resulting poses were analyzed according to both visual inspection and by the computed docking scores available in the Autodock Tools GUI.

### Crystal Structure Determination of the Apo-Form of *Streptococcus pyogenes* GapN

Crystals of the apo-enzyme (WT and C284S mutant) were grown at 20°C by the vapor diffusion method in hanging drops. The WT apo-enzyme crystals were grown by mixing 1 μl protein (WT) at a concentration of 15 mg/ml in 1 × PBS (pH 7.4) with 1 μl mother liquor containing 0.2 M Li_2_SO_4_, 0.1 M Bis-Tris pH 5.5 and 25% PEG 3350. Crystals of the C284S variant were obtained at a protein concentration of 2.5 mg/ml, by again mixing 1 μl of the protein solution in 1 × PBS (pH 7.4) with 1 μl of reservoir containing 0.2 M Li_2_SO_4_, 0.1 M Bis-Tris pH 6.5 and 18% PEG 3350. Crystals grew either as plates or prisms in 4–5 days.

Diffraction data were collected at the ESRF (Grenoble, France) for the wild-type apo-enzyme on beam line ID30-B on a Dectris Pilatus3 6 M detector at a wavelength of λ = 0.918401 Å and for the C284S variant on beam line ID23-2 at a wavelength of λ = 0.873128 Å on a Dectris Pilatus3 × 2 M detector. All diffraction data were collected at a temperature of 100 K after adding 25% glycerol as cryo-protectant. The collected data sets were indexed, integrated and scaled with XDS and the resulting scaling was analyzed with the program Aimless (CCP4). As Aimless detected significant anisotropy in the data sets, the data generated with XDS were reprocessed with Staraniso^1^ with an < I/σI > cutoff of 1.2.

The apo-structure of wild-type GapN was solved by molecular replacement using Phaser MR with the apo-enzyme from *S. mutans* (PDB entry: 1EUH) as a search model. Subsequent molecular replacement calculations for the C284S GapN mutant used a tetramer of the initial *S. pyogenes* apo-structure as a search model. The structures were initially refined with Refmac 5 (CCP4) using NCS restraints, Babinet scaling and isotropic temperature factor refinement and extensive rebuilding in Coot. Subsequently, coordinates, B-factors, occupancies, and TLS-parameters were refined with Buster after adding hydrogens with Hydrogenate.

### Statistical Analysis

The number of biological replicates (n) and the tests used to determine statistical significance for each data set are indicated in the respective figure captions. Statistical analyses were performed using GraphPad Prism 8 software.

## Results

### GapN Is the Major NADPH Source in *Streptococcus pyogenes*

We hypothesized that the GapN mediated reaction is the major source of NADPH in *S. pyogenes*. Since NADPH is needed in numerous anabolic reactions, e.g., fatty acid and amino acid biosynthesis, it is an essential cofactor. In bacteria, NADPH is usually derived from the oxidative part of the pentose phosphate pathway. Since the enzymes of the oxPPP are not encoded in the genome of *S. pyogenes*, the GapN mediated conversion of G3P to 3-PG might represent the major NADPH generating reaction in these bacteria. This hypothesis is supported by the observation that either GapN or the oxPPP are missing in the majority of sequenced streptococci available at the KEGG database with only a subset of organisms containing both pathways (Table 3).

**TABLE 3:** Presence (+) or absence (−) of the oxPPP and GapN in streptococcal species.

Species	GapN	oxPPP
*S. oralis*	+	+
*S. pneumoniae*	+	+
*S. sanguinis*	+	+
*S. cristatus*	+	+
*S. pyogenes*	+	−
*S. agalactiae*	+	−
*S. mutans*	+	−
*S. thermophilus*	+	−
*S. equi*	+	−
*S. uberis*	+	−
*S. parauberis*	+	−
*S. dysgalactiae*	+	−
*S. gallolyticus*	+	−
*S. salivarius*	+	−
*S. infantarius*	+	−
*S. iniae*	+	−
*S. equinus*	+	−
*S. anginosus*	−	+
*S. intermedius*	−	+
*S.macedonicus*	−	+
*S.gordonii*	−	+
*S.pseudopneumoniae*	−	+
*S.suis*	−	+
*S.mitis*	−	+

**FIGURE 1:**
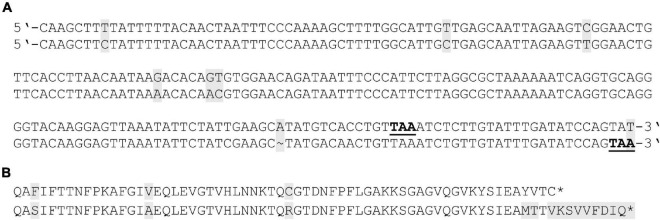
Resequencing of the *gapN* gene of *S. pyogenes* M49 strains NZ131 and 591. **(A)** 3′-end of the *gapN* gene sequence as determined in this work for the NZ131 and 591 strains (lower lines) in comparison to the NCBI derived sequence of the NZ131 strain (upper lines). **(B)** Amino acid sequences of the N-terminus of GapN for the NZ131 and 591 strains according to the resequencing in this work (lower line) in comparison to the NCBI derived sequence for NZ131 (upper line). Stop codons are indicted in bold underlined letters **(A)** or asterisks **(B)**. Discrepancies in sequences are indicated by gray backgrounds.

To further validate this hypothesis, we analyzed the cofactor specificity of GapN and the conventional GapDH of *S. pyogenes* M49 strain 591. For that purpose, both genes were ligated into vectors of the pASK-IBA series for heterologous expression and subsequent purification via StrepTactin based affinity chromatography. In this context, we re-sequenced the *gapN* genes of the *S. pyogenes* M49 strains NZ131 (GeneBank: OK337836) and 591. A BLASTnt analysis revealed significant differences in our sequence data in comparison to the NCBI derived *gapN* sequence of the NZ131 strain (Figure 1). Most importantly, the sequence derived in this study shows a frameshift in comparison to the NCBI derived sequence, leading to an open reading frame extension of 24 bp and, consequently, to a protein with 8 additional amino acid residues at its amino-terminus. Apart from this, several other differences were found in the 3′-end of the gene, leading to differences in amino acid residues at five positions near the C-terminus of the protein as compared to the NCBI-derived sequence. In BLASTp analyses, the resulting protein shows a slightly higher similarity to GapN proteins of other sequenced *S. pyogenes* serotype strains (99.2–99.7%) as compared to the one based on the NCBI derived sequence (98.6–99.1%). According to our analysis, there are no differences between the *gapN* gene sequences of the NZ131 and 591 strains. The reference sequence meanwhile available for strain 591 is identical to the sequence we determined.

To determine the state in which GapN can be found while in solution, we used size-exclusion chromatography coupled to multi-angle light scattering. GapN (496 aa) eluted at approximately 14 ml, revealing a calculated weight-average molar mass of 2.022 × 10^5^ Da (± 2.494%) (Supplementary Figure 1). Inserting our sequence in Expasy ProtParam revealed a molecular weight of 52937.55 Da per monomer or 2.118 × 10^5^ Da for a tetramer. The determined molar mass and the molecular weight of MALS and ProtParam are in very good agreement under the assumption that the enzyme is present in solution as a tetramer.

The purified GapN and GapDH of *S. pyogenes* M49 strain 591 were analyzed for their cofactor specificity. Therefore, the enzymatic activity of both enzymes was measured in the presence of NAD^+^ as well as NADP^+^. Purified GapN showed a specific activity of 33.6 ± 9.3 U/mg protein (*n* = 4) in the presence of NADP^+^, while no activity was observed in the presence of NAD^+^. In contrast, GapDH was completely inactive in the presence of NADP^+^, while with NAD^+^ a specific activity of 21.1 ± 5.5 U/mg (*n* = 4) was detected. This indicates that GapN is highly specific for NADP while GapDH cannot utilize NADP at all. The Km values of the purified GapN were determined to be 0.36 mM (± 0.05 standard error) for NADP and 0.58 mM (± 0.19 standard error) for DL-G3P.

**FIGURE 2:**
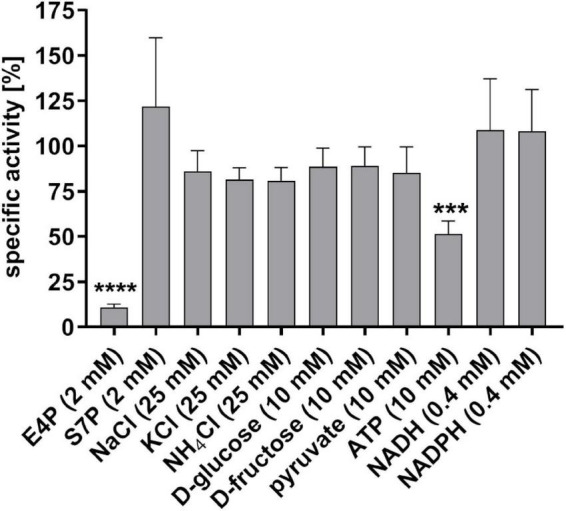
Influence of different substances on the specific activity of purified *S. pyogenes* GapN. Specific activities are shown relative to the specific activity in a standard assay mix. E4P, erythrose 4-phosphate; S7P, sedoheptulose 7-phosphate, mean values and standard deviations; *n* = 4, one-way ANOVA, ***p < 0.001, ****p < 0.0001.

Furthermore, we analyzed the impact of different salts, sugars and metabolic intermediates on the specific activity of the purified *S. pyogenes* GapN. All substances tested were previously described to affect the activity of GapN in *S. mutans* (Crow and Wittenberger, 1979). The respective substances were added to the standard GapN activity assay in the concentrations indicated in Figure 2. Of the 11 substances tested, only E4P and ATP significantly reduced the activity of the *S. pyogenes* GapN by 90 and 50%, respectively, with the other substances displaying no significant impact.

### GapN Is Essential in *Streptococcus pyogenes* and Other Streptococci Lacking the Oxidative Part of the Pentose Phosphate Pathway

**FIGURE 3:**
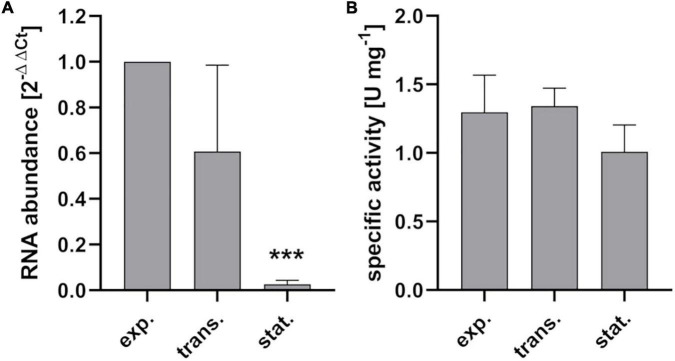
Growth phase associated gene expression and enzyme activity of GapN. **(A)** *gapN* transcript abundance and **(B)** specific GapN activity in crude extracts of *S. pyogenes* during exponential (exp.), transition (trans.) and stationary (stat.) growth phases in CDM-LAB medium. Mean values and standard deviations, ***p < 0.001, *n* = 3, pairwise comparison with exponential phase using paired *t*-test.

During growth of *S. pyogenes* M49 strain 591 in chemically defined medium for lactic acid bacteria (CDM-LAB), the *gapN* transcript is present in all growth phases (Figure 3A). The transcript abundance is highest during exponential growth, stays relatively stable in the transition phase but strongly drops in the stationary phase of the bacteria (Figure 3A). Correspondingly, specific GapN activity is detectable in all growth phases (Figure 3B). In contrast to the transcript abundance, however, the specific activity does not decrease significantly toward the stationary phase, hinting at a high stability of the enzyme.

**FIGURE 4:**
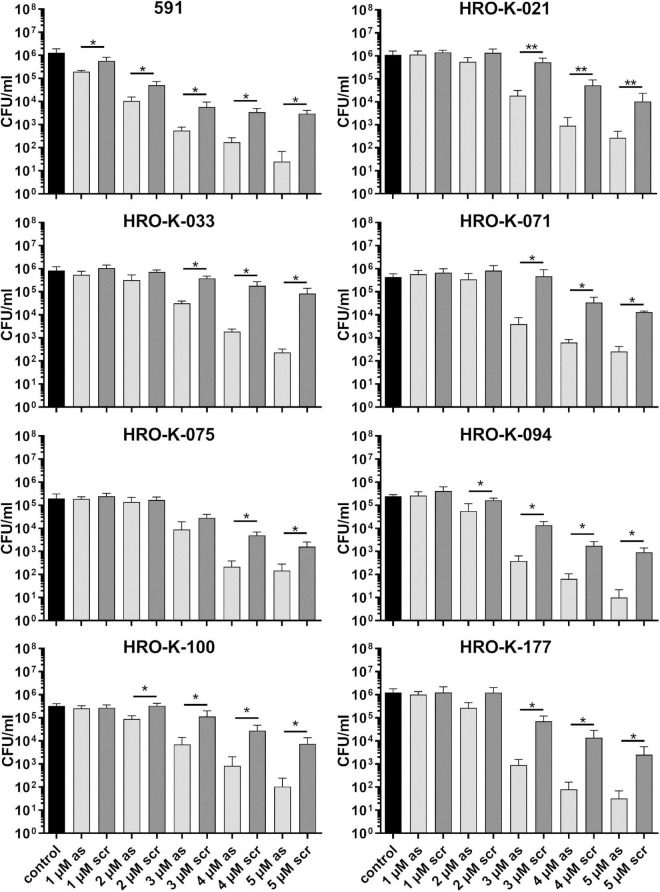
Survival of different *S. pyogenes* strains in the presence of (RXR)_4_XB-gapN-asPNA. Mean values and standard deviations, *n* = 5 (HRO-K021) or *n* = 4 (all other strains), *p < 0.05, **p < 0.01, pairwise comparison via Mann-Whitney *U*-test.

**FIGURE 5:**
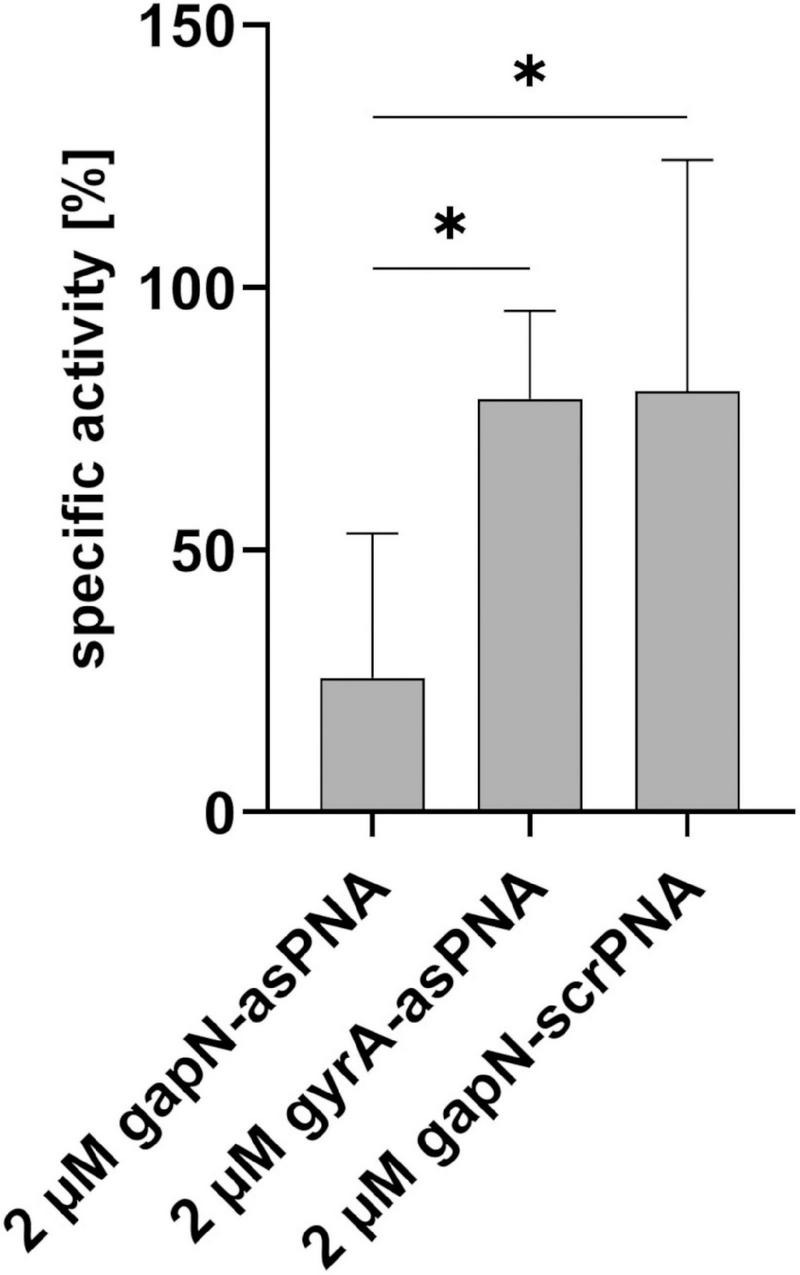
Influence of PNA treatment on the specific GapN activity. GapN activity in protein crude extracts of *S. pyogenes* M49 treated with either 2 μM (RXR)_4_XB-gapN-asPNA, (RXR)_4_XB-gyrA-asPNA or (RXR)_4_XB-gapN-scrPNA (scr) in comparison to an untreated control. Mean values and standard deviations, *n* = 3, *p < 0.05, unpaired two-tailed *t*-test.

Next, we analyzed whether a knock-down of GapN can affect the viability of *S. pyogenes*. For this purpose, the M49 laboratory strain 591 and seven macrolide-resistant clinical *S. pyogenes* isolates (Table 1), together representing eight different *emm* types and four different FCT-types, were subjected to kill assays with *gapN*-specific asPNA. The *gapN* asPNA were complementary to the *gapN* start codon region. As a control, water as well as scrambled (scr) PNA were used. Both asPNA and scrPNA were fused to the cell penetrating peptide (CPP) with the sequence (RXR)_4_XB (R—arginine, X—6-aminohexanoic acid, B—beta alanine), as it has previously been described that this CPP facilitates the uptake of PNA into *S. pyogenes* (Barkowsky et al., 2019). For all eight strains a significant reduction of the viable counts was achieved upon treatment with *gapN* asPNA (Figure 4 and Table 1). The scrambled control PNA also reduced the viable counts, indicating that the cell penetrating peptide exhibits cell toxicity. The effect of the asPNA was, however, significantly higher than the effect of the scrambled controls in all tested strains. Upon treatment with 5 μM *gapN* asPNA the viable counts of all strains were between one and two orders of magnitude lower than upon treatment with the scrambled control PNA. Already upon treatment with 2 μM of *gapN* specific asPNAs for 6 h, the specific GapN activity in protein crude extracts of *S. pyogenes* M49 strain 591 was significantly reduced to about 25% as compared to the untreated control, whereas the scrambled control PNA or PNA targeting the essential gyrase subunit A encoding *gyrA* gene did not reduce GapN activity significantly (Figure 5).

**FIGURE 6:**
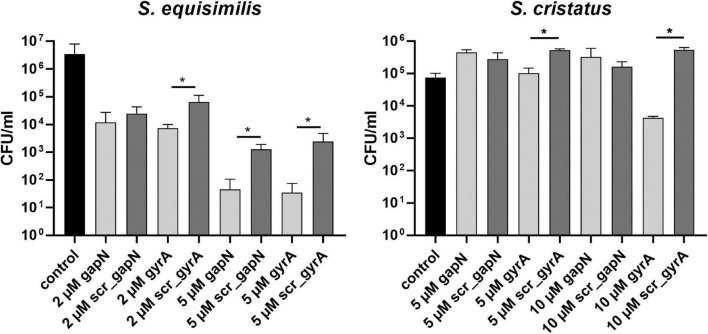
Survival of different streptococcal species in the presence of (RXR)_4_XB-PNA directed against *gapN* or *gyrA*. Mean values and standard deviations, *n* = 4 (all PNAs) or *n* = 8 (controls) *p < 0.05, pairwise comparison with corresponding scrPNA using Mann-Whitney *U-*test.

Additionally, we tested the effect of *gapN* asPNA treatment on two further streptococcal species. *S. dysgalactiae* ssp. *equisimilis* resembles *S. pyogenes* in as it also lacks the oxPPP but possesses a GapN. *S. cristatus* possesses both GapN and the oxPPP. For *S. cristatus* and *S. dysgalactiae* ssp. *equisimilis*, asPNA treatment has not been described previously. However, using asPNA targeting *gyrA* we could show that (RXR)_4_XB is a suitable CPP to mediate PNA uptake for both species as *gyrA* asPNA treatment reduced the viable counts significantly in comparison to the scrambled control PNA (Figure 6). In accordance with the hypothesis that GapN is only essential in species lacking the oxPPP, the viable counts of *S. dysgalactiae* ssp. *equisimilis* were significantly reduced upon treatment with *gapN* asPNA, while this was not the case for *S. cristatus* (Figure 6).

**FIGURE 7:**
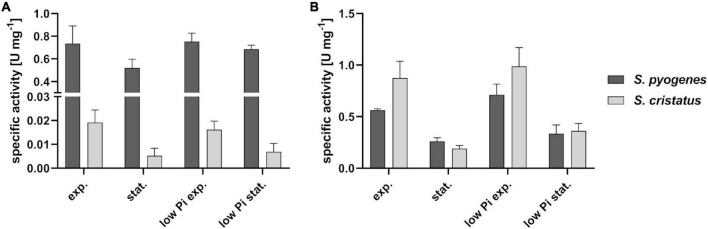
Phosphate concentration and growth phase associated specific GapN activity in *S. pyogenes* and *S. cristatus*. Activity of **(A)** GapN and **(B)** GapDH in protein crude extracts of *S. pyogenes* M49 and *S. cristatus* during exponential (exp.) and stationary (stat.) phase of growth in CDM-LAB (42 mM phosphate) resp. CDM-LAB low phosphate (1 mM phosphate; low Pi) media. Mean values and standard deviations, *n* = 3.

Consequently, we checked whether GapN is active in *S. cristatus* at all. Therefore, the growth phase associated specific GapN activity of *S. cristatus* growing in CDM-LAB was analyzed and compared to *S. pyogenes* 591. As can be seen in Figure 7A, GapN activity can be detected in crude extracts from *S. cristatus*, but the activity is far lower than the activity detected in crude extracts of *S. pyogenes* in the exponential and stationary phases. In contrast, the specific GapDH activity in the crude extracts was roughly the same in *S. cristatus* and *S. pyogenes* in both growth phases (Figure 7B).

It has been proposed previously that in *S. pyogenes* GapN is needed to keep glycolysis running under low phosphate conditions, as the GapN-mediated conversion of G3P to 3-PG does not consume inorganic phosphate, in contrast to the GapDH and phosphoglycerate kinase mediated reactions (Levering et al., 2012). Therefore, GapN activity in crude extracts of *S. pyogenes* and *S. cristatus* grown in CDM-LAB low phosphate conditions was analyzed. However, the strongly reduced phosphate supply (1 mM in comparison to 42 mM in the conventional CDM-LAB) did not lead to an increased GapN activity in either species (Figure 7).

### The High Resolution Crystal Structure of GapN in the Apo-State Provides a Starting Point for Structure-Based Drug Design

At present, no structures of GapN from *S. pyogenes* have been reported. The closest homolog for which crystal structures have been determined is the enzyme from *S. mutans*, which shares 86% sequence identity with *S. pyogenes* GapN. This enzyme, as well as more distantly related homologs from *Bacillus halodurans* (58% identity), *Methanocaldococcus janaschii* (37% identity) and *Escherichia coli* (35% identity), is present as a homotetramer with D2 symmetry in crystal structures. To further characterize the function of the *S. pyogenes* GapN and provide a starting point for future screening campaigns, we determined the crystal structure of the GapN by molecular replacement using the apo-form of an *S. mutans* tetramer as a search model. The structure was refined at a resolution of 2 Å against data derived from triclinic crystals containing two tetramers in the asymmetric unit (PDB: 7PKJ) (Table 4). The structure revealed a covalent modification of the enzyme at the active site residue C284, which could be modeled as β-mercaptoethanol although this compound was not present during purification. Hence, the enzyme must have been modified following heterologous expression in *E. coli*. To eliminate this modification and generate a true apo-state of the enzyme, the C284S variant was generated and crystallized. Its crystal structure was also determined by molecular replacement, with a tetramer of the wild-type protein as search model. The variant again crystallized in the P1 space group, but in this case the unit cell contained four tetramers and the structure was refined at 1.5 Å resolution (PDB entry 7PKC). Due to its higher resolution, the structure of the C284S mutant is presented and discussed here.

**TABLE 4:** Data collection and refinement statistics.

	Apo-structure of wild-type *S. pyogenes* GapN	Apo-structure of C284S mutant of *S. pyogenes* GapN

Data collection		

Space group	P1	P1
**Cell dimensions**		
*a*, *b*, *c* (Å)	97.75, 99.89, 106.18	97.67, 126.04, 148.39
α, β, γ (°)	77.67, 75.19, 67.12	96.36, 104.81, 84.23
Resolution (highest shell) (Å)	48.15–1.99 (2.17–1.99)	47.63–1.50 (1.52–1.50)
Diffraction best/worst direction after cut-off (Å)	1.989/3.378	1.499/3.481
Anisotropic ΔB (Å^2^)	17.23	3.36
^a^ *R*_*sym*_	0.304 (1.002)	0.122 (0.705)
^b^ *R*_*pim*_	0.213 (0.655)	0.087 (0.497)
*CC_1/2_*	0.847 (0.396)	0.992 (0.466)
^c^< *I*/σ*I* >	3.195 (0.943)	5.444 (1.569)
Completeness (sphere;%)	0.629 (0.137)	0.685 (0.149)
Completeness (ellipse;%)	0.875 (0.707)	0.900 (0.638)
Redundancy	2.91 (3.23)	2.76 (2.89)
**Refinement**		
Resolution (highest shell) (Å)	20.03–1.99 (2.08–1.99)	19.97–1.50 (1.58–1.50)
No. reflections (highest shell)	153,157 (2,918)	744,550 (14,532)
^d^*R*_*work*_/^e^*R*_*free*_	0.1923/0.2319 (0.2930/0.3204)	0.1999/0.2316 (0.2734/0.2842)
No. of atoms	30,879	63,495
Protein	28,364	56,829
Water	2,243	6,361
Ions SO_4_^2–^/PO_4_^3–^	90	155
Glycerol	182	150
*B*-factors (Å^2^)	24.99	16.85
Protein (best/worst chain)	21.97 (20.68/32.51)	15.02 (13.42/22.02)
Water	27.39	24.57
Ions SO_4_^2–^/PO_4_^3–^	72.48	45.08
Glycerol	49.47	54.06
^f^Ramachandran statistics (%)	96.24/3.31/0.45	96.49/3.09/0.42
**RMS deviations in**		
Bond lengths (Å)	0.008	0.008
Bond angles (°)	1.01	1.01
Torsion angles (°)	14.77	14.13
Planar groups (Å)	0.0121	0.0135

*^a^R_sym_ = Σ_hkl_Σ_i_ | I_i_—< I > | /Σ_hkl_Σ_i_I_i_ where I_i_ is the i^th^ measurement and < I > is the weighted mean of all measurements of I.*

*^b^R_pim_ = Σ_hkl_1/(N-1) ^1/2^ Σ_i_| I_i_(hkl)—I (hkl) |/Σ_hkl_Σ_i_I(hkl), where N is the redundancy of the data and I (hkl) the average intensity.*

*^c^ < I/σI > indicates the average of the intensity divided by its standard deviation.*

*^d^R_work_ = Σ_hkl_ | | F_o_| –| F_c_| |/Σ_hkl_| F_o_| where F_o_ and F_c_ are the observed and calculated structure factor amplitudes.*

*^e^R_free_ same as R for 5% of the data randomly omitted from the refinement. The number of reflections includes the R_free_ subset.*

*^f^Ramachandran statistics were calculated with Coot. Numbers in parentheses refer to the respective highest resolution data shell in each dataset.*

**FIGURE 8:**
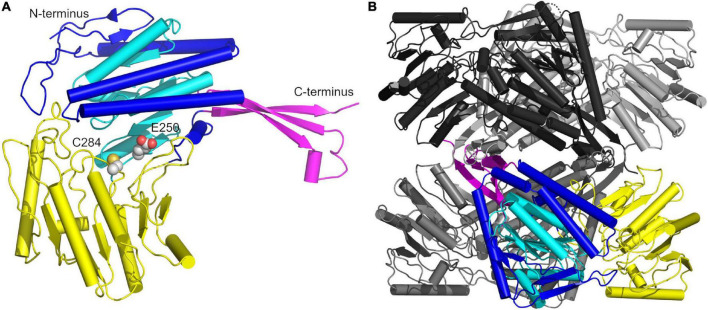
Overview of the crystal structure of the *S. pyogenes* GapN C284S mutant. **(A)** Architecture of the monomer with the protruding domain colored in magenta, the cofactor binding domain in blue encompassing the Rossmannoid fold (cyan), and the catalytic α/β domain in yellow. The catalytically active C284 and E250 are shown in CPK representation. **(B)** The GapN tetramer with one monomer colored as in **(A)** and the other three monomers in black, light gray and dark gray.

The GapN-monomer from *S. pyogenes*, like its counterparts from other organisms can be subdivided into three domains (Figure 8A; Cobessi et al., 1999). Starting at the N-terminus, residues 2–118, 145–252, and 450–464 form the cofactor binding domain containing a Rossmannoid fold (residues 145–252, cyan with the remainder of the domain in blue) which harbors five β-strands and three α-helices and mediates binding to NADP (Hanukoglu, 2015). The two other domains are the protruding domain composed of residues 119–144 and 464–475 (magenta), which is critical for oligomerization, and the catalytic α/β domain encompassing residues 253–445 (yellow). The catalytically active cysteine, which is responsible for the nucleophilic attack on the carbonyl carbon of G3P, is located at position 284, in close proximity to E250, the other key catalytic residue. E250 interacts with a water molecule, which deprotonates the cysteine in the deacylation step of the NADPH generating mechanism (Cobessi et al., 2000). The monomers assemble into a tetramer displaying D2 symmetry which is formed by two interlacing dimers with dimer-dimer contacts primarily mediated by the protruding domain, while contacts within each dimer are formed by a β-sheet of the protruding domain interacting with a β-sheet of the catalytic α/β domain via salt bridges as well as α-helices (residues 232–251) of the Rossmannoid fold of each chain (Figure 8B).

**FIGURE 9:**
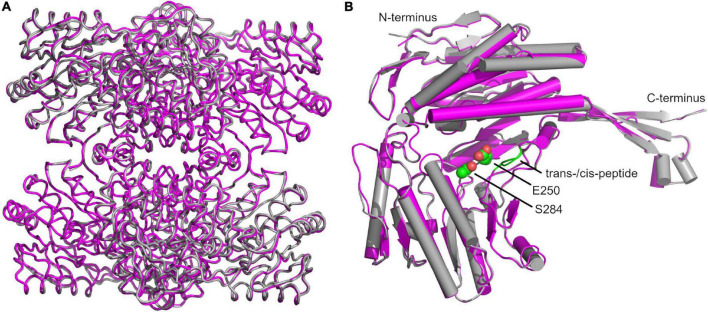
Superimposition of structures of the *S. pyogenes* C284S GapN mutant and *S. mutans* GapN. **(A)** Comparison of the apo-forms of the GapN tetramers from *S. mutans* (PDB: 1EUH) in gray and *S. pyogenes* (C284S variant) in magenta. **(B)** Comparison of monomers with the cis-peptide bond between residues G438 and T439 indicated in green as well as the catalytic residues S284 (in lieu of C284) and E250 highlighted in CPK representation with C-atoms in green.

A superimposition of our structure with that of GapN from *S. mutans* (PDB: 1EUH) resulted in a root mean square deviation (RMSD) of 0.61 Å for the tetramer and 0.41 Å, if only the respective A-chains were aligned (Figure 9A). The comparison revealed an unexpected difference for residues G438 and T439 (Figure 9B). In all chains of the wild-type and C284S variant a cis-peptide was observed while only half of the chains in the reference structure from *S. mutans* were modeled in the cis configuration. Considering that the formation of a cis-peptide outside an X-P (X denoting any amino acid) peptide is rather rare, the electron density maps of our structures and the published structures were critically examined (Jabs et al., 1999). This analysis confirmed the formation of a cis-peptide bond in both *S. pyogenes* as well as in *S. mutans*. As this peptide bond is within 11.2 Å of the active site, it might be relevant for the proper function of the enzyme. Furthermore, in the more distantly related *Bacillus halodurans* GapN, a cis-peptide can be observed in all chains between G447 and P448 (PDB: 3PRL), further underscoring the importance of this feature.

### Homology Modeling Facilitates Prediction of GapN Inhibitors

**FIGURE 10:**
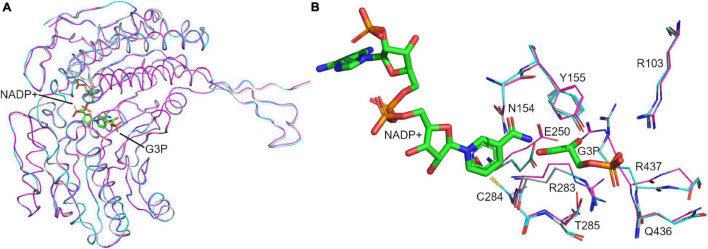
Modeled structure of holo GapN from *S. pyogenes*. Superimposition of the crystal structure of the apo GapN (C284S mutant) (magenta), the homology-modeled holo GapN from *S. pyogenes* (cyan) and the modeling template crystal structure from *S. mutans* (gray). **(A)** Aligned backbones of the monomers are shown in ribbon representation, showing their very high similarity. **(B)** Aligned binding-site residues, showing some differences in side-chain positions. The cofactors, G3P and NADP^+^, are shown in stick representation with carbons colored green.

To pave the way for future drug screening assays, we generated a structure of the tetrameric holo-enzyme by homology modeling before the crystal structures for *S. pyogenes* GapN were available. Due to its high sequence-identity of 86%, GapN from *S. mutans* was chosen as the template protein for the model. There are four structures of this protein available in the RCSB protein data bank, two of which, 2ESD and 1QI1 (D’Ambrosio et al., 2006), consist of the holo-enzyme with the physiological substrate, G3P, bound. Holo-enzymes were preferred, because the catalytic site would have the necessary conformational changes to accommodate the substrate and possible competitive inhibitors. Both the latter structures have mutations in the active site: C284T (PDB: 1QI1) and E250A (PDB: 2ESD). We found that by using the SWISS-MODEL webserver the conformations of the catalytic residues were appropriately rebuilt when using the 1QI1 structure as the template (Figure 10). On the other hand, when the 2ESD structure was used as the template, E250 adopted a conformation which did not align to the reported “intermediate” or “inside” conformations (Munoz-Clares et al., 2011). Hence, we chose 1QI1 as the structural template. Assessment of the resultant homology model using statistical criteria computed by the SWISS-MODEL server indicated a high certainty in the generated model (Supplementary Figure 2). Aligning the model to the template structure in PyMol resulted in a remarkably low RMSD of 0.081 Å.

From our models and the literature on NAD(P)-dependent aldehyde dehydrogenases (Munoz-Clares et al., 2011), the catalytic cysteine (C284) can either adopt a “resting”-conformation, in which the thiol points away from the ligand, or an “attacking”-conformation in which it points toward the aldehyde moiety of the substrate. E250 is also involved in the catalytic cycle in which it activates water molecules and can either adopt an “inside” or “intermediate” conformation, in which it points, respectively, toward the binding site or in the opposite direction.

To assess the quality of the generated model and to provide a benchmark for evaluating future inhibitor candidates, the native ligand G3P was re-docked to GapN using both GLIDE and Autodock4.2. Re-docking with GLIDE was successful in reproducing the binding pose observed in the 1QI1 structure after manually rotating C284 to the “resting” conformation for the docking assay. The best pose achieved a docking score of –7.7 kcal/mol and featured mainly favorable interactions between the ligand and the protein. Aligning the generated ligand pose to the structure of G3P from the crystal structure 1Q1 resulted in an RMSD of 1.08 Å. The only small difference between this pose and the template structure was in the position of the aldehyde oxygen. GLIDE did not reproduce its interaction with N154. Instead, the aldehyde oxygen formed a hydrogen-bond to T285 while its interaction with C284 was successfully reproduced. It should also be noted that the restrained energy-minimization routine in Maestro leads to the loss of the hydrogen-bond of the aldehyde-oxygen of G3P to N154 present in the original crystal structure. The best results from docking G3P to a structure with C284 in the “attacking” conformation resulted in docking scores between –7.6 and –7.3 kcal/mol but showed an alternative positioning of the hydroxyl-group of G3P. In all of these slightly worse-scoring poses, the hydroxyl-group interacted with T285 rather than with R437 (as in 1QI1). Re-docking in Autodock resulted in a similar pose and a docking score of –7.3 kcal/mol.

A comparison of the crystal structure of the apo-enzyme and the homology model of the holo-enzyme (which, as stated before, was built before the crystal structure of the apo-enzyme was determined) revealed a very high degree of similarity between the two and alignment in PyMol (DeLano, 2002) resulted in an RMSD of 0.345 Å (Figure 10A). The structures differed mainly in the binding site, where the absence of G3P appeared to result in an alternative conformation of R437 which is similar to that observed in apo-structures of GapN from *S. mutans* that lack a ligand: PDB 1EUH and 2ID2 (Figure 10B). However, the *cis*-peptide bond between G438 and T439 observed in the crystal structure of the apo-enzyme was not present in the homology model of the holo-enzyme or its structural template from *S. mutans.* It is unclear whether this cis-peptide bond arises because of the missing ligands in the apo-form of the protein.

**FIGURE 11:**
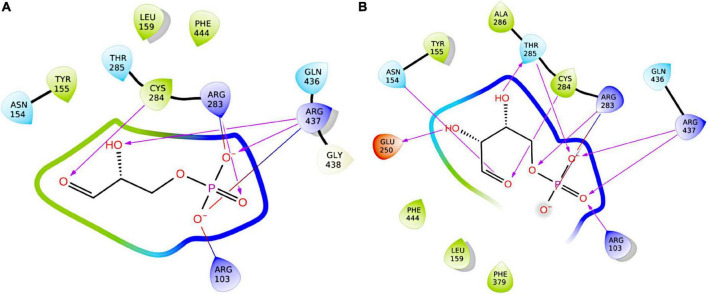
Two-dimensional representations of the docking poses of ligands to GapN. Protein-ligand hydrogen-bonds are represented by arrows in the direction of the acceptor. Salt-bridge interactions are indicated by lines colored red at the negative charge and blue at the positive charge. **(A)** Best pose of re-docked G3P to GapN. **(B)** Highest-scoring pose of E4P.

Crow and Wittenberger (1979) purified the template protein from *S. mutans* and identified both E4P and sedoheptulose-7-phosphate (S7P) to have significant inhibitory effects on its catalytic activity, with E4P having greater inhibitory activity than S7P. To assess the quality of our structural model, we used computational docking algorithms to predict the binding modes and affinities of the two compounds for *S. pyogenes* GapN. While E4P was predicted by both GLIDE and Autodock to bind to the enzyme with a binding affinity comparable to G3P: –7.6 kcal/mol (GLIDE; Figure 11), –7.2 kcal/mol (Autodock), both algorithms predicted no inhibitory effect of S7P: ∼–5 kcal/mol (GLIDE); –0.19 kcal/mol (Autodock), whose binding was sterically hindered.

Docking G3P to the *S. mutans* GapN template structure 1QI1 after manually mutating T284 back to the wild-type cysteine resulted in a pose with the same score of –7.6 kcal/mol (GLIDE). E4P was docked with a slightly higher affinity score for the template protein of –8.1 kcal/mol while S7P was predicted to have a similarly low affinity for the binding site of roughly –4 kcal/mol (GLIDE). Docking S7P to 1QI1 with Autodock resulted in a slightly higher affinity for the *S. mutans* GapN than for the modeled *S. pyogenes* GapN of roughly –1.4 kcal/mol.

## Discussion

GapN related enzymes occur not only in bacteria but also in archaea, fungi and plants (Habenicht, 1997; Iddar et al., 2005), however, the physiological importance of this enzyme is not entirely clear in most organisms. It is likely though that the GapN mediated reaction contributes to the NADP/NADPH metabolism. NADPH is an essential cofactor in numerous anabolic reactions such as in the biosynthesis of fatty acids, desoxyribonucleotides and, e.g., proline and glutamic acid, two amino acids *S. pyogenes* is prototrophic for (Levering et al., 2016; Xu et al., 2018). In bacteria, the major NADPH supply for most species are the two oxidizing reactions in the pentose phosphate pathway (Spaans et al., 2015). This sugar degrading pathway, however, is absent in some bacteria, e.g., in several streptococcal species. In such species, the GapN mediated reaction is likely to be the major source for the production of NADPH.

Essentiality of GapN has previously been proposed for *S. pyogenes* on the basis of a high-throughput transposon mutant library screening approach for the identification of essential genes (Le Breton et al., 2015). This is in line with our data, as survival of *S. pyogenes* laboratory and clinical strains was significantly reduced upon asPNA-based inhibition of GapN production. We observed a significant specific reduction of viable bacteria by *gapN* asPNA at concentrations above 1–4 μM, depending on the *S. pyogenes* strain. These concentrations are in the same range as previously published for asPNA treatment against well-known essential genes in *S. pyogenes* and other gram-positive bacteria. (RXR)_4_XB coupled *gyrA*-specific asPNA were reported to significantly reduce *S. pyogenes* viable counts at concentration ≥ 4 μM in the same experimental setup as used in our study (Barkowsky et al., 2019). In *Staphylococcus aureus*, asPNA against *fmhB* and *gyrA* resulted in impaired growth at concentrations of 10 μM (KFF)_3_K (Nekhotiaeva et al., 2004), and minimal inhibitory concentrations of 6.25–12.5 μM were described for PNAs against *rpoD*, respectively, depending on the CPP used for PNA translocation into the cells (Bai et al., 2012). Moreover, (RXR)_4_XB-coupled anti *rpoA* PNA were effective in *Listeria monocytogenes*, as indicated by minimal inhibitory concentrations of no more than 4 μM against clinical isolates (Abushahba et al., 2016). Hence, the *gapN*-specific asPNA concentrations needed to significantly reduce viable counts of *S. pyogenes* are in the range reported for other essential genes of gram-positive bacteria.

In accordance with the proposed role of GapN in NADPH supply in species lacking the oxPPP, *S. dysgalactiae* spp. *equisimilis* was equally prone to asPNA based GapN inhibition demonstrating the essentiality of GapN in this species as well. As expected, *S. cristatus* as a species carrying both oxPPP and *gapN*, was unaffected by the *gapN* specific asPNA. This species generally is susceptible to asPNA treatment, as upon treatment with *gyrA* specific asPNA viable counts were significantly reduced. Moreover, we were able to establish (RXR)_4_XB as a suitable CPP for PNA administration in *S. cristatus* and *S. dysgalactiae* spp. *equisimilis*, for which—to the best of our knowledge—no CPP facilitating uptake of PNA was described before.

As an essential gene, GapN should be constantly present in *S. pyogenes.* In growth phase dependent transcriptome and proteome analyses of *S. pyogenes* M49 NZ131, Chaussee et al. (2008) found the *gapN* transcript in both exponential and stationary phases of growth, with a (non-significant) 2-fold increase of transcript abundance in the stationary phase (Chaussee et al., 2008). Furthermore, they detected two isoforms of GapN in the proteome with a threefold and ninefold increase in the stationary phase in comparison to the exponential phase of growth (Chaussee et al., 2008). In another DNA microarray-based transcriptome study by Beyer-Sehlmeyer et al. (2005), no differential expression of *gapN* was detected when comparing growth phases during growth in THY (Beyer-Sehlmeyer et al., 2005).

The increased relative abundance in the proteome could not be verified by our enzyme activity data, as we found relatively similar GapN activities in cells from both exponential and stationary growth phases. Additionally, our qPCR data showed a significant decrease of the *gapN* expression in the stationary phase, which is contradictory to the data of Beyer-Sehlmeyer et al. (2005) and Chaussee et al. (2008). Although the stationary phase sampling time points in these studies differ from our sampling time point (18 h and 16 h vs. 7 h in our study), the reason for the discrepancies between the results obtained here and the published data remains unclear. However, an analysis of the *gapN* expression in *S. equinus*—a species lacking the oxPPP—by Asanuma and Hino (2006) shows that it rather resembles the findings of our work, as the authors found an eightfold decrease in *gapN* transcript abundance compared with the exponential growth phase (Asanuma and Hino, 2006). The authors argue that *gapN*-expression is dependent on the NADPH requirement. During exponential growth, NADPH is highly needed due to the high rate of reductive biosynthesis in rapidly growing cells. Upon reaching the stationary phase, growth ceases and the need for NADPH decreases, leading to a reduction of *gapN*-expression (Asanuma and Hino, 2006). This hypothesis was also supported by their finding that *gapN* transcription is enhanced by CcpA, which also regulates *ldh*-expression. The LDH level, as an indicator of fermentation rate, would also rise when the reductive biosynthesis rises due to an increased growth rate. Asanuma and Hino (2006) furthermore detected that GapN activity drops by a factor of 32 in the stationary phase of *S. equinus*. Although our gene expression data are consistent with those described by Asanuma and Hino (2006), we did not detect significantly reduced GapN activity in the stationary phase in *S. pyogenes*. It can be speculated that the *S. pyogenes* enzyme has a higher stability than the *S. equinus* enzyme, however, both enzymes are relatively similar and have an amino acid sequence homology of about 85%. Nevertheless, all studies showed that either the *gapN* transcript or the protein itself or the specific enzyme activity were found in all growth phases, which is consistent with the essentiality of GapN.

Among the streptococcal GapN enzymes, the one from *S. mutans* has been characterized in the most detail. The *S. pyogenes* enzyme shares a high sequence similarity of 86% with the *S. mutans* enzyme. Accordingly, the structural properties of the two enzymes are rather similar, but not identical. Both enzymes form a homotetramer and contain a Rossmanoid fold for NADP/H binding (Cobessi et al., 1999, 2000; Marchal et al., 2001). Our X-ray crystallographic analysis revealed that apo-GapN from both *S. mutans* and *S. pyogenes* contains an unusual cis-peptide not involving a proline. This cis-peptide is located approximately 11 Å from the G3P binding pocket of the *S. pyogenes* apo-enzyme and might therefore influence substrate binding.

In accordance with our data for *S. pyogenes* GapN, *S. mutans* GapN also exclusively uses NADP as a cofactor (Crow and Wittenberger, 1979). While most enzymes of the aldehyde dehydrogenase (ALDH) family rely on NAD as a cofactor, both GapN from *S. mutans* and from *S. pyogenes* possess a binding pocket for NADP, with the residues R209, K177 and T180 interacting with the 2′-phosphate group, mediating the specificity for NADP. ALDH enzymes using NAD instead of NADP, such as the non-phosphorylating GapDH of *Thermoproteus tenax*, contain an isoleucine instead of a threonine in this binding pocket, leading to a lower affinity for NADP (Brunner et al., 1998).

In *S. mutans*, inhibitory effects on GapN were observed for E4P, S7P and phosphohydroxypyruvate (Crow and Wittenberger, 1979). E4P and phosphohydroxypyruvate were also described to inhibit GapN extracted from pea shoots, maize leaf and the green algae *Chlamydomonas reinhardtii* (Kelly and Gibbs, 1973; Iglesias et al., 1987). E4P and S7P are produced in the non-oxidative part of the PPP (Stincone et al., 2015), which is present in *S. pyogenes*. Thus, they could play a role in the *in vivo* regulation of the enzyme. Our structure-based *in silico* docking to *S. pyogenes* and *S. mutans* GapN predicted E4P to have a similar or higher binding affinity as compared to the natural substrate G3P, thus endowing it with inhibitory activity.

This prediction correlates with our experimental data as the specific GapN activity was significantly decreased to 10.8 ± 1.9% in the presence of 2 mM E4P. While E4P as a metabolite is not an appropriate candidate for a therapeutic GapN inhibitor, the experimental data on E4P inhibition of GapN underline the accuracy of the predictions of the *in silico* docking analysis. Furthermore, in accordance with the model predictions, S7P does not inhibit *S. pyogenes* GapN. The binding site residues that interact with G3P are perfectly conserved in *S. pyogenes* GapN and *S. mutans* GapN and align very well in the structural models. Both structures have a narrow binding site which might sterically hinder binding of the larger S7P compared to the smaller E4P and G3P. The docking results indicate that the previously observed inhibition of *S. mutans* GapN by S7P might require structural rearrangements of the protein, which are not accounted for in the docking performed here, or arise from allosteric effects.

Our measurements show that another inhibitory regulator of *S. pyogenes* GapN is ATP. ATP is generated during glycolysis, which provides most of the energy in *S. pyogenes*. As a glycolytic enzyme, GapN could be downregulated by an excess of ATP. Likewise, GapN from *S. mutans* and *Sulfolobus solfataricus*, a thermophilic archaeon, were also reported to be inhibited by ATP to some extent (Crow and Wittenberger, 1979; Ettema et al., 2008). Other described stimulators and inhibitors of *S. mutans*, as well as compounds from the carbohydrate metabolism that could influence the GapN activity, showed no significant effects on the activity of the *S. pyogenes* enzyme.

Although it should have no effect on the results for inhibition and thus model validation as well as cofactor specificity, it is worth mentioning that our purified enzymes show a lower activity than already published values. For example, Iddar et al. (2003) described an activity of 143.2 U/mg for a purified GapN from *S. pyogenes* heterologously expressed in *E. coli*, whereas we only found a specific activity of 33.6 ± 9.3 U/mg. This might be the effect of the different purification methods. Iddar et al. (2003) purified GapN based on a protocol involving protein precipitation, DEAE- cellulose-, phenyl- sepharose-, and hydroxyapatite-chromatography. In contrast, we added a StrepTag to the N-terminus of GapN and purified the enzyme via StrepTactin affinity chromatography. However the K_*m*_ values determined for G3P and NADP with our GapN preparation resembled those published by Iddar et al. (2003), indicating a sufficient amount of functional enzyme in our preparations.

In summary, we have shown that GapN is essential in *S. pyogenes.* Our structural model of the *S. pyogenes* GapN correctly predicts the experimentally generated inhibition data. Hence, in future work, it should be possible to identify selective inhibitors for GapN that might be developed into lead compounds for new antimicrobial drugs to address the problem of increasing antibiotic resistance in *S. pyogenes*.

## Data Availability Statement

The datasets presented in this study can be found in online repositories. The names of the repository/repositories and accession number(s) can be found below: https://www.ncbi.nlm.nih.gov/genbank/, OK337836; http://www.wwpdb.org/, 7PKJ; and http://www.wwpdb.org/, 7PKC.

## Author Contributions

PE: formal analysis, investigation, validation, visualization, writing—original draft preparation, and writing—review and editing. LA: investigation, data curation, and writing—review and editing. JT: investigation, validation, visualization, and writing—review and editing. EZ, CM, JJ, CS, and LG: investigation and writing—review and editing. NB: investigation. AN-A: supervision and writing—review and editing. BK: resources and writing—review and editing. HS: supervision, data curation, validation, and writing—review and editing. RW: conceptualization, supervision, and writing—review and editing. TF: conceptualization, project administration, supervision, validation, visualization, writing—original draft preparation, and writing—review and editing. All authors contributed to the article and approved the submitted version.

## Conflict of Interest

The authors declare that the research was conducted in the absence of any commercial or financial relationships that could be construed as a potential conflict of interest.

## Publisher’s Note

All claims expressed in this article are solely those of the authors and do not necessarily represent those of their affiliated organizations, or those of the publisher, the editors and the reviewers. Any product that may be evaluated in this article, or claim that may be made by its manufacturer, is not guaranteed or endorsed by the publisher.

## Footnotes

1
http://staraniso.globalphasing.org/cgi-bin/staraniso.cgi

## Abbreviations

3-PG: 3-phosphoglycerate
asPNA: antisense PNA
CDM-LAB: chemically defined medium for lactic acid bacteria
CPP: cell penetrating peptide
E4P: erythrose 4-phosphate
G3P: glyceraldehyde 3-phosphate
GapN: non-phosphorylating glyceraldehyde-3-phosphate dehydrogenase (EC 1.2.1.9)
GapDH: glyceraldehyde-3-phosphate dehydrogenase (EC 1.2.1.12)
oxPPP: oxidative part of the pentose phosphate pathway
PNA: peptide nucleic acid
RMSD: root mean square deviation
S7P: sedoheptulose 7-phosphate.
