# Supplementary material for: The Non-phosphorylating Glyceraldehyde-3-Phosphate Dehydrogenase GapN Is a Potential New Drug Target in Streptococcus pyogenes
Source: Front Microbiol. 2022 Feb 15;13:802427. doi: 10.3389/fmicb.2022.802427 (PMC8886048; doi:10.3389/fmicb.2022.802427)
Supplement: Supplementary file 1 [file Data_Sheet_1.docx]

Supplementary Material

# Supplementary Figures and Tables

## Supplementary Tables

**Supplementary Table 1.** Overview of quality controls of PNA used in this study

| **ID** | **Name** | **Sequence** | **Purity [%]** | **[M+H]^+^_measured_ [Da]** | **[M+H]^+^ _calculated_  [Da]** |
| --- | --- | --- | --- | --- | --- |
| S818-C1 | (RXR)_4_XB-anti-gapN-Spyo | R-Ahx-RR-Ahx-RR-Ahx-RR-Ahx-R-Ahx-bA-x-ttgccaacgt | 98.877 | 4732.502 | 4732.427 |
| S818-D1 | (RXR)_4_XB-scr-gapN-Spyo | R-Ahx-RR-Ahx-RR-Ahx-RR-Ahx-R-Ahx-bA-x-catgtgctac | 98.430 | 4732.449 | 4732.427 |
| S788-A2 | (RXR)_4_XB-anti-gapN-Scri | R-Ahx-RR-Ahx-RR-Ahx-RR-Ahx-R-Ahx-bA-x-cacgtgacac | 98.155 | 4726.402 | 4726.439 |
| S788-B2 | (RXR)_4_XB-scr-gapN-Scri | R-Ahx-RR-Ahx-RR-Ahx-RR-Ahx-R-Ahx-bA-x-ccccggaaat | 98.219 | 4726.533 | 4726.439 |
| S767-H1 | (RXR)4XB-anti-gyrA Spyo | R-Ahx-RR-Ahx-RR-Ahx-RR-Ahx-R-Ahx-bA-x-tgcatttaag | 98.777 | 4771.392 | 4771.438 |
| S708-C2 | (RXR)_4_XB-anti-gyrA-Scri | R-Ahx-RR-Ahx-RR-Ahx-RR-Ahx-R-Ahx-bA-x-cttgcattaa | 98.065 | 4731.657 | 4731.431 |
| S708-D2 | (RXR)_4_XB-scr-gyrA-Scri | R-Ahx-RR-Ahx-RR-Ahx-RR-Ahx-R-Ahx-bA-x-tagtactact | 97.744 | 4731.585 | 4731.431 |
| S689-A2 | (RXR)4XB-anti-gapN-Sequ | R-Ahx-RR-Ahx-RR-Ahx-RR-Ahx-R-Ahx-bA-x-ttgtcaacgt | 98.698 | 4747.377 | 4747.426 |
| S689-B2 | (RXR)4XB scr-gapN-Sequ | R-Ahx-RR-Ahx-RR-Ahx-RR-Ahx-R-Ahx-bA-x-tcagtcagtt | 97.594 | 4747.469 | 4747.426 |

X = Aminohexanoic acid (Ahx), B = beta Alanine (bA), eg = 8-amino-3,6-dioxaoctanoic (x) (short PEG-Spacer)

## Supplementary Figures





**Supplementary Figure 1. GapN tetramerizes in solution.** Determination of the oligomeric state of GapN by SEC-MALS. The numbers on the left indicate the calculated molecular masses. The MALS result are shown as particle mass (dots) and elution volume via the refractive index [RI] concentration signal (continuous lines).


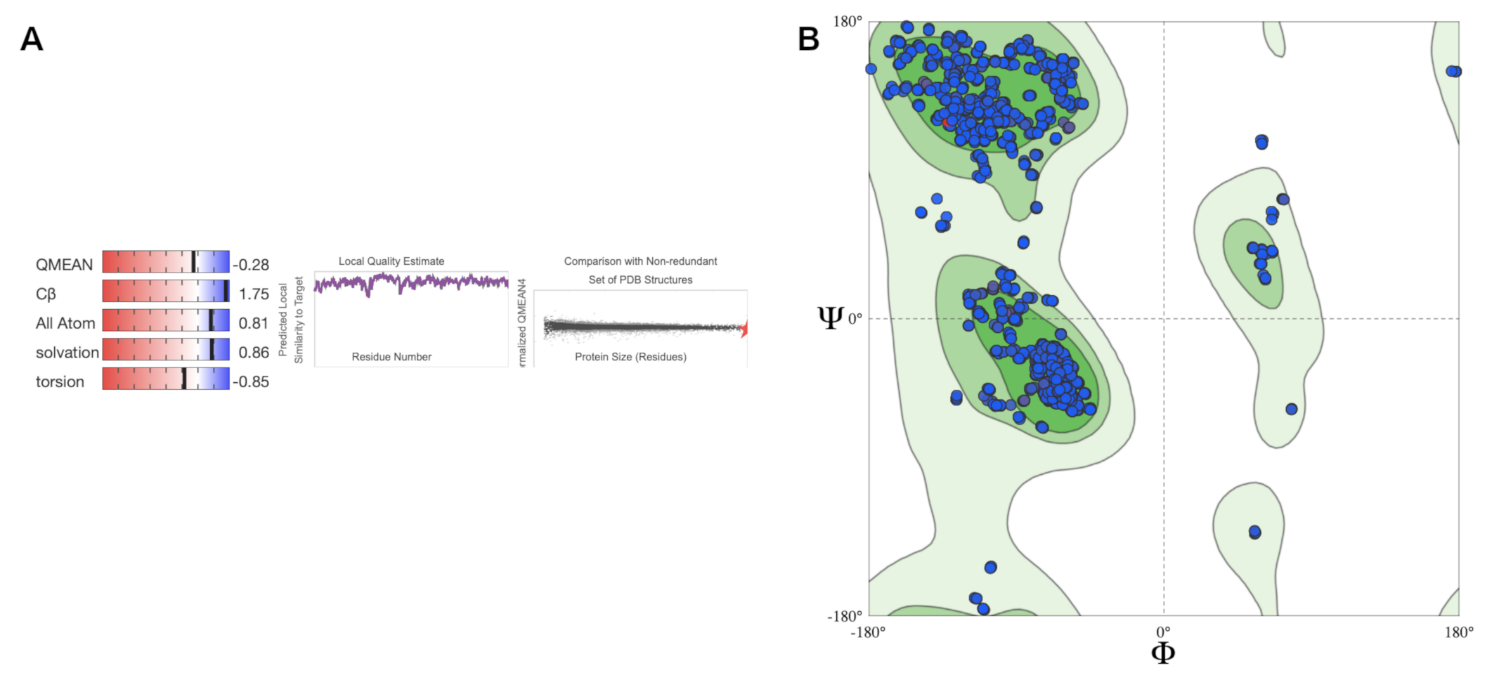


**Supplementary Figure 2. Homology Modeling with SWISS-MODEL using the structure (PDB: 1QI1) of the holo-form of *S. mutans* GapN generated a high-quality model of the holo-form of *S. pyogenes* GapN** Model parameters, local quality and statistical comparison indicate a high, overall quality (**A**). Ramachandran plot of the resulting model (all residues) (**B**).
